# Mapping Transmission Risk of Lassa Fever in West Africa: The Importance of Quality Control, Sampling Bias, and Error Weighting

**DOI:** 10.1371/journal.pone.0100711

**Published:** 2014-08-08

**Authors:** A. Townsend Peterson, Lina M. Moses, Daniel G. Bausch

**Affiliations:** 1 Biodiversity Institute, University of Kansas, Lawrence, Kansas, United States of America; 2 Department of Tropical Medicine, Tulane School of Public Health and Tropical Medicine, New Orleans, Louisiana, United States of America; Division of Clinical Research, United States of America

## Abstract

Lassa fever is a disease that has been reported from sites across West Africa; it is caused by an arenavirus that is hosted by the rodent *M. natalensis*. Although it is confined to West Africa, and has been documented in detail in some well-studied areas, the details of the distribution of risk of Lassa virus infection remain poorly known at the level of the broader region. In this paper, we explored the effects of certainty of diagnosis, oversampling in well-studied region, and error balance on results of mapping exercises. Each of the three factors assessed in this study had clear and consistent influences on model results, overestimating risk in southern, humid zones in West Africa, and underestimating risk in drier and more northern areas. The final, adjusted risk map indicates broad risk areas across much of West Africa. Although risk maps are increasingly easy to develop from disease occurrence data and raster data sets summarizing aspects of environments and landscapes, this process is highly sensitive to issues of data quality, sampling design, and design of analysis, with macrogeographic implications of each of these issues and the potential for misrepresenting real patterns of risk.

## Introduction

Lassa fever (LF) is a zoonotic disease caused by Lassa virus (LASV), a member of the *Arenaviridae* family [1]. Clinical manifestations range from mild febrile illness to severe vascular leakage, hemorrhage, shock, and death. Introduction of LASV into humans occurs through direct or indirect contact with excreta of the natural reservoir, the rodent *Mastomys natalensis*, although precise modes of transmission are not well characterized [2]. Human-to-human transmission of LASV through contact with blood and other bodily fluids has been documented, particularly in clinical settings [3].

Although decades of experience and numerous epidemiological studies make it clear that LF is a phenomenon of the West African sub-region [1], [4], the details of the spatial distribution of LASV and LF remain unclear. Understanding the incidence and distribution of LF has been hampered by lack of easily-available diagnostics and limited public health surveillance infrastructure in the region [5]. LF is best characterized in areas with research programs focusing on the disease, particularly central and southern Nigeria and eastern Sierra Leone [5], [6]. Beyond these focal areas of surveillance activity, estimates of LASV distribution are coarse, providing little basis for inference into intervening areas of West Africa. As a result, the reported incidence of LF shows significant spatial clustering owing to reporting bias, a common phenomenon among neglected and emerging diseases.

**Figure 1:**
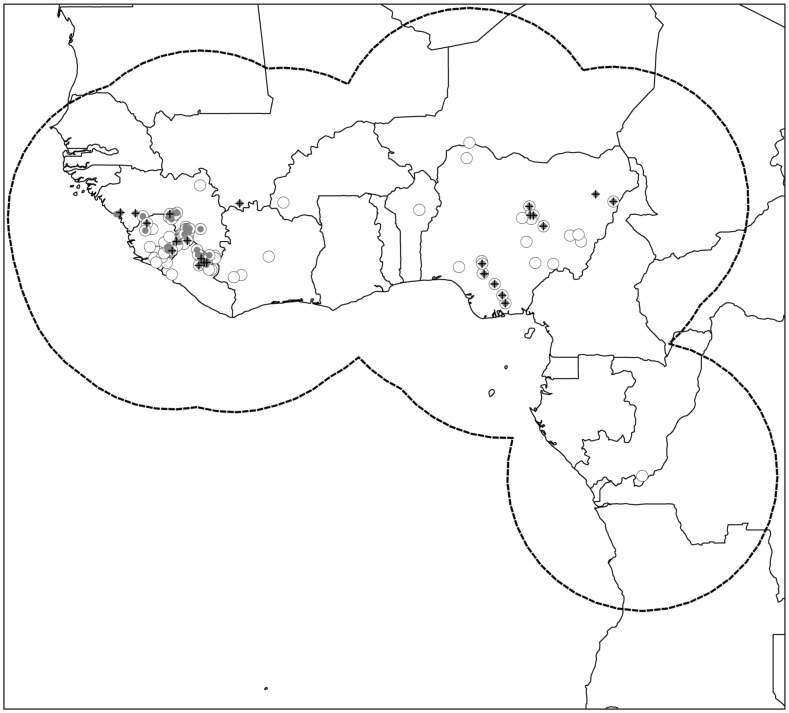
Summary of occurrence data input in the ecological niche models. The dashed outline shows the limits of the area of analysis, with circles indicating the data used by Fichet-Calvet and Rogers [7]. Gray circles indicate data that met quality control criteria levels 1 and 2 (see Table 1). Black crosses indicate data after random subsampling (see Methods for rationale and details).

**Figure 2:**
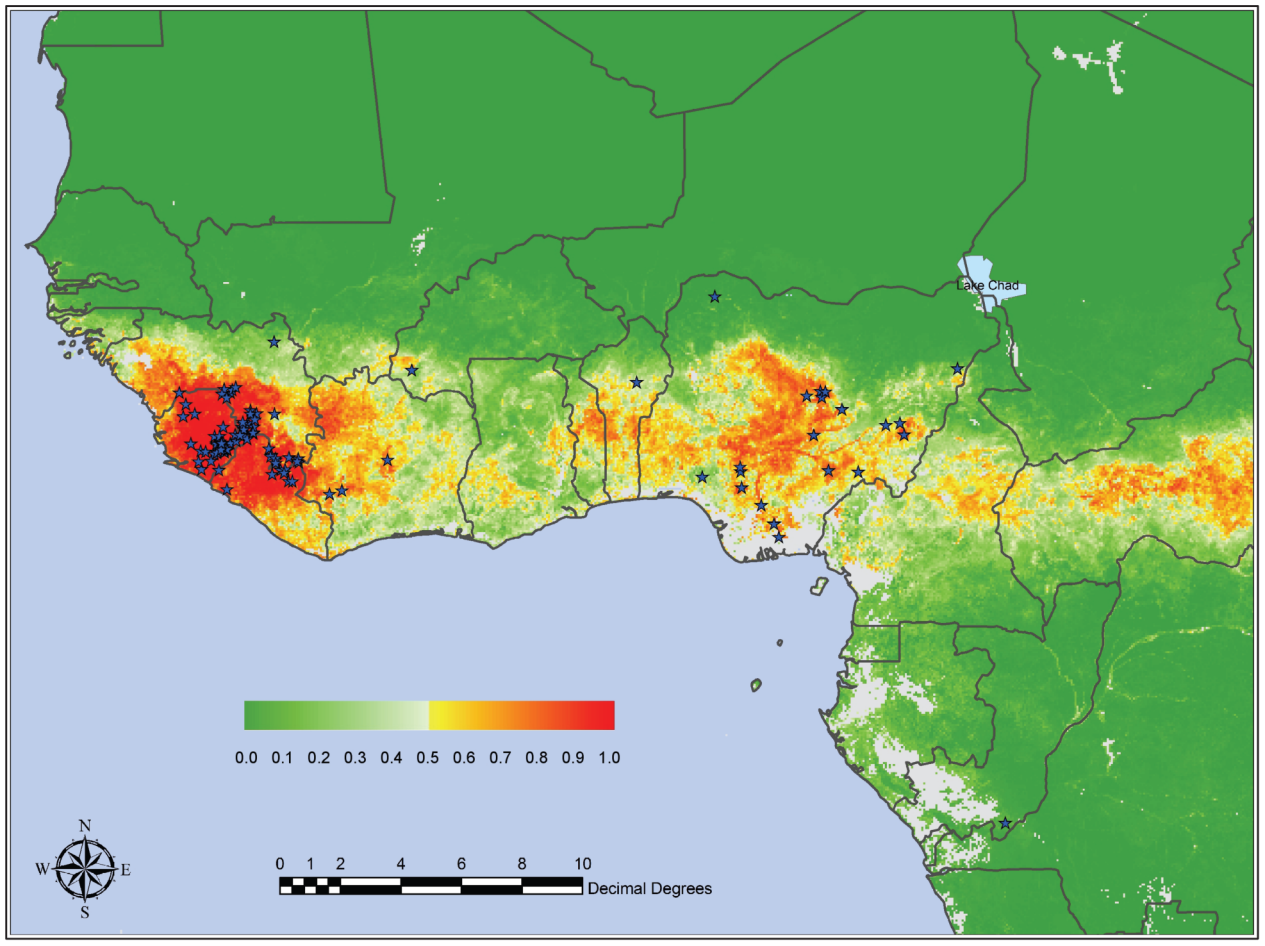
Mean predicted LF risk map from the Model 2 series developed by Fichet-Calvet and Rogers [7], with posterior probability color scale from 0.0 (no risk) to 1.0 (highest risk) shown at inset. Gray areas are areas either lacking suitable imagery (because of cloud contamination—coastal Nigeria and Cameroon) or that are so distant in environmental space that predictions were not possible. Used with permission.

In a recent publication, Fichet-Calvet and Rogers [7] provided “risk maps of Lassa fever in West Africa,” assembling a data set of 111 occurrences of LASV infection and LF from published seroprevalence studies and clinical case reports (Figure 1). Rodent occurrence data were also compiled in the study, but were excluded from analyses. The resultant maps have been cited and republished frequently as definitive distribution risk maps for LF [8]. The maps, however, developed using ecological niche modeling approaches, show several characteristics of concern (Figure 2) [9]: high-risk areas are broadly disjunct at the western and eastern extremes of West Africa, without apparent coincidence with known biogeographic or environmental breaks, and (most worrisome) high-risk areas coincide closely with areas of most intense sampling (i.e., near research centers). This result suggests either that LF occurrence has been sampled thoroughly in the only areas where it is most prevalent (i.e., its distribution is well-characterized by existing sampling), or that models were overfit to input data, producing risk maps with little generality or predictive power [10], offering a falsely clear geographic picture of risk. Overfit models are those that replicate well the input data, identifying areas that have already been sampled, but that have little generality that might permit genuine prediction and anticipation of risk in areas where sampling has not occurred. Such models often perform poorly when challenged to predict independent sets of data, as might be produced in the present case via intensive on-ground studies in, for example, Benin or Togo.

While the patterns presented by Fichet-Calvet and Rogers may indeed be correct, the concerns that their results engender demand more in-depth examination. Here, we present a series of further analyses of their data, re-examining them in terms of possible sources of bias in risk-map development (see parallel example in [11]). In particular, we address three possible biases in the occurrence data and analytical methods related to (1) quality control regarding definition of occurrence (i.e., high versus low-confidence documentation of LASV infection/LF cases), (2) controlling for sampling bias (i.e., accounting for the fact that some areas have been subject to intensive surveillance for LF while others have not), and (3) balancing potential for Type 1 and Type 2 errors appropriately in resulting models (i.e., taking into account the fact that sites of known occurrences *versus* sites with no known occurrence have different levels of confidence associated with them). Throughout, our focus is on macrogeographic implications of biases—if potential sources of confusion are random, their effects might simply diffuse any signal that does exist in the data; if, however, these sources of confusion have consistent environmental correlates, then resulting risk maps will incorporate these biases and macrogeographic implications will manifest. It should be noted that the goal and result of our analysis is not necessarily to present more accurate risk maps for LF *per se*, but rather to illustrate pitfalls inherent in risk mapping when data are not carefully considered and controlled.

## Methods

### Input data and study area

The occurrence data presented in Table 1 of Fichet-Calvet and Rogers [7] were captured in spreadsheet format and organized for further analysis. This data set was carefully referenced, which permitted us to reexamine the quality of diagnostics for each occurrence record as part of our analyses (see below). A few records were excluded from our analyses for lack of access to documents referenced, leaving an initial 107 of Fichet-Calvet and Rogers' 111 data records as inputs to our analyses. We refer henceforth refer to this data set as the ‘raw’ data.

**Table 1:** Quality control schema for diagnosis confidence for Lassa fever occurrence data from Fichet-Calvet and Rogers [7].

Validity rating	Description
1	Virus isolate, PCR-positive, or ELISA-positive *Mastomys natalensis*
2	Virus isolate, PCR-positive, ELISA-positive, or plaque neutralization assay-positive human case of LF
3	Human case of LF supported by IFA or other laboratory test result
4	Serosurvey positive based on ELISA or plaque neutralization assay
5	Serosurvey positive based on IFA or other laboratory test
6	Human case of LF without supporting laboratory test
7	No evaluable information, no original data, or simply no data

Note that when multiple ratings applied to single points in space, we assigned the higher of the ratings, assuming that the greatest confidence is the most appropriate.

Abbreviations: ELISA, enzyme-linked immunosorbent assay; IFA, immunofluorescent assay; PCR, polymerase chain reaction.

The occurrence data were analyzed in the context of environmental variation across West Africa, focusing on the region relatively close to sites of known LF cases as a study region. We examined occurrence data to identify the largest spatial disjunction (∼800 km), and used a buffer of this radius around known occurrences as our study region (Figure 1). By choosing this region as the area within which models are to be calibrated, we assume it to have been accessible to LASV and its rodent host for potential colonization, and that some possibility exists of LF cases in the region being detected, diagnosed, and reported. These assumptions are not inconsequential; in effect, we have assumed that within this region LF cases should be detected and reported, and that sites and environments represented within this area that do not hold case reports have a higher probability of lacking such records because the conditions are not appropriate for LASV transmission and maintenance [12].

### Effects of quality control of diagnoses

The degree of confidence of a diagnosis of LASV infection/LF is impacted by various factors; many or even most patients with LF present with non-specific manifestations extremely difficult to distinguish from many other common febrile illnesses in West Africa, such as malaria or typhoid fever, making clinical diagnosis difficult and laboratory diagnosis imperative [1], [13], [14]. Unfortunately, no FDA-approved or widely validated laboratory tests exist for LF, leaving only a few laboratories in the world capable of reliably providing the diagnosis using various “in-house” assays. Over the past few decades, only Sierra Leone, Nigeria, and Guinea have been able to conduct laboratory tests for LF in-country, and these often only intermittently and at times with significant questions regarding quality control [4], [13], [15]. Furthermore, the various assays employed for LF have varying degrees of sensitivity and specificity [1], [4]. Tests that directly indicate presence of LASV, such as cell culture, PCR, ELISA antigen assays, and plaque neutralization assays [16], [17], provide the most definitive proof of infection, whereas antibody tests such as immunofluorescence assays are generally less specific and thus yield lower confidence in the diagnosis [18].

Uncertainty may also exist in the attribution of the geographic location of LASV infection; infected persons may travel during the incubation period (which may be up to three weeks), potentially resulting in incorrect attribution of occurrence to site of illness rather than infection [19]. It should be noted that *M. natalensis* are ubiquitous in rural areas of West Africa, and that specific infecting events are rarely recognized by persons with LF. Infection is thought most often to occur via unwitting exposure to *M. natalensis* excreta. Even when human-to-human transmission is involved, the specific contact or infecting event is usually not recognized, again creating uncertainty regarding the geographic origin of the case.

To add a measure of quality control and to quantify the confidence that should be accorded to each LF occurrence record, we subset the Fichet-Calvet and Rogers data according to reliability of the diagnostic method for each LF case record; an expert (DGB) with long experience with LF, rated each record according to a 7-point scale of certainty of diagnosis (Table 1) that ranged from laboratory confirmed LASV-positive rodents (level 1, highest confidence) to reports on human cases providing no information about the basis of the diagnosis of LF (level 7, lowest confidence). We accorded the highest confidence to rodent-derived records owing to the lower probability of long-distance movements of rodents that could result in attribution of LASV occurrence in nonrepresentative areas. We excluded serosurveys (i.e., studies in which healthy populations were tested for LASV-reactive antibodies), since the timing, and thus geographic location of the exposures of positive cases, could not be known. Serosurveys are also fraught with uncertainty based on non-specificity of the antibody assays, cross-reactive antibodies, and issues regarding setting cut-offs for a positive result [1]. This quality control schema was used to compare models based on all records (levels 1-7) with models based on only levels 1 and 2, where confidence of the presence of LASV infection/LF was highest.

### Effects of uneven sampling across space

A second contrast explored was between the raw data set and a subset in which we attempted to remove effects of spatial concentrations of LF cases created by intensive sampling. Concentrations of case reports may result from intensive transmission and genuinely high incidence in the region, but may also simply reflect areas of intensive study and sampling. To this end, we identified the area of southeastern Sierra Leone, southern Guinea, and northwestern Liberia as representing areas of artificial over-reporting based on the intensive studies of LF that have focused on these regions [4], [13], [20], [21]. We identified 44 LF cases (41% of the raw data set) coming from these intensively studied regions and randomly removed 75% of them to produce a reduced data set that included only 11 occurrences from these areas. We then compared models based on the full raw and reduced data sets. Finally, owing to interesting results that came from this procedure (see below), we created 10 such randomized subsamplings and examined variation among models based on each, in effect testing to assure that the particular subsample chosen in our initial reduction did not present spurious characteristics not representative of the broader data set.

### Effects of balance of error types

Finally, we examined effects of relative balancing of two types of error in model calibration on model results. Any spatial prediction manifests two types of error: omission error, in which known presences of species (in this case, LASV-infected rodents or humans) are left out of the prediction, and commission error, in which areas not known to hold the species are predicted as suitable [22]. Occurrence data are peculiar in that presence data are relatively strong in their confidence—i.e., although some may represent misdiagnoses or mistaken geographic references, the great bulk of presence records accurately link the occurrence of a given species with a particular site on landscapes. In contrast, confidence in absence of occurrences is much lower [9]. In the case of LF, absence of occurrence data *may* represent a genuine lack of LASV transmission in a region. However, occurrence data may be absent even when LASV is present in an area because (1) no humans are present to get infected, (2) no contact occurs between humans and the rodent reservoir to result in LASV transmission, (3) humans are infected but no laboratory facility is available to make a diagnosis, or (4) humans are diagnosed but cases are not reported or case data is not available to researchers. In some of the aforementioned circumstances, risk of LF may be high despite the absence of recognized occurrences. As a consequence, presence data and its associated omission error should be accorded much higher weighting in model calibration than absence data and its associated commission error [9].

To this end, we compared two approaches to model calibration; in our first approach, we weighted omission and commission errors equally to replicate the error balance used by Fichet-Calvet and Rogers [7]. We combined multiple independent model optimizations of the niche modeling algorithm GARP, which depend on separate random subsamplings of available occurrence data and on distinct random-walk model optimizations. Because GARP optimizes a parameter that balances the two error components equally [23], these consensus models reflect the equal error weighting situation. In our second approach, we prioritized minimization of omission error over minimization of commission error by using the “best subsets” procedure of Anderson *et al*. [24] that applies and prioritizes a filter based on omission error rates before a filter based on commission error rates. Comparisons of models created with these two approaches provide an effective illustration of the effects of equal *versus* the more appropriate omission-weighted error balance schemes in model calibration.

### Model evaluation

Because data documenting LF occurrences across West Africa are relatively few and are highly uneven in their distribution, our evaluation of the effects of the three potential biases explored in this paper was in large part qualitative and visual. We compared views of model outputs before and after correcting for these potential biases, as well as with the original figures presented by Fichet-Calvet and Rogers (Figure 2). Niche models, such as those that both we and Fichet-Calvet and Rogers used, are fitted in environmental spaces [9] and thus manifest effects of biases only to the degree that these factors create consistent, non-random associations in environmental dimensions. If effects of a potential bias are negligible or manifested on local scales only, then differences before and after corrections for this bias would be manifested as ‘salt and pepper’ or randomly dispersed and not as consistent spatial differences.

For each of the three potential biases we considered, we created difference maps contrasting both the magnitude and spatial topology of the differences between the two treatments. Again, we were seeking areas of consistent departure as opposed to salt-and-pepper. Because initial model results showed intriguing patterns, we sought to explore model results in the Sierra Leone-Liberia-Guinea area in greater detail. We therefore carried out the 10 replicate subsamplings of available occurrence data described above, which allowed us to assess whether our particular initial subsampling yielded nonrepresentative results. Again, our assessment was largely qualitative, as the results were quite clear in showing consistent results from model to model.

As a local-scale evaluation of the import of our model predictions, for this same region, we analyzed rodent-based detections of LASV specifically. We used sources listed in Table 1 of Fichet-Calvet and Rogers [7] that were not included in their analysis and additional data from rodent surveys in 13 Sierra Leonean villages collected in 2009–2010, where 117 of 460 (25.4%) *Mastomys* were PCR-positive for an arenavirus (L. Moses and D. Bausch, in prep.). We overlaid LASV prevalences in rodents on the model predictions to assess whether explanatory power existed at finer spatial scales. Because rodent sampling was fairly restricted in its spatial extent, we related prevalence of LASV in rodents to predicted suitability on the overall LF map (i.e., the one in which all three biases described above had been corrected), and evaluated the relationship between the two quantities using linear regression.

## Results

### Effects of correcting for potential bias

#### Quality control

**Figure 3:**
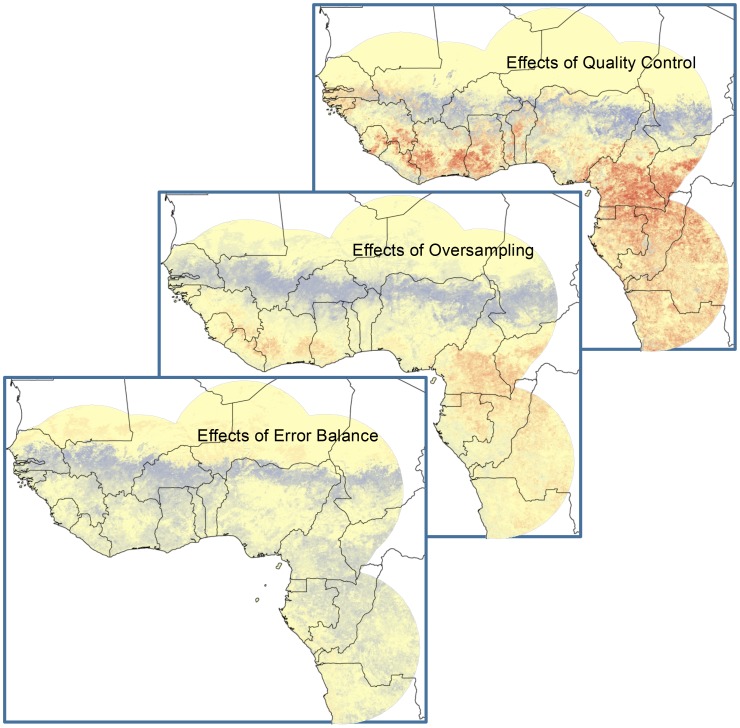
Summary of effects of three factors assessed in this study as potentially influencing model outcomes: quality control of input occurrence data (top panel), reduction of oversampling of occurrences in certain areas (middle), and weighting omission *versus* commission errors appropriately (bottom). In each case, the map represents a difference between our corrected and our mimicking of the original analysis such that a score of 100 (dark red) indicates a situation wherein the original analysis overemphasized the suitability of a site, whereas a score of -100 (dark blue) indicates underemphasis. All three maps are shown on the same color scale.

The original 107 case-occurrence points we considered reduced to only 20 when only high-confidence points (i.e., levels 1 and 2) were considered. Comparing models based on these two data sets (Figure 3), models without occurrence data quality control overemphasized humid areas of West and Central Africa, at the expense of areas in the Sahel that may also be suitable for LF occurrence. However, areas of contrast between the two maps were spatially autocorrelated—that is, they were highly contiguous, such that quality control of diagnoses of LF cases had clear and consistent implications for the ecological niche model that resulted and the potential distribution that was reconstructed.

#### Reducing effects of oversampling

Of the original 111 LF case-occurrence points considered by Fichet-Calvet and Rogers [7], 44 came from southeastern Sierra Leone, southern Guinea, and northwestern Liberia. To avoid over-representing of these intensively sampled areas, we discarded 75% (33 occurrences) based on occurrence point densities elsewhere (i.e., comparing with Nigeria). As with the previous factor, this manipulation caused clear, contiguous, and spatially autocorrelated macrogeographic effects that were more or less parallel to those in the quality control manipulation (Figure 3). Again, humid areas of West and Central Africa were overemphasized by models for which sampling density was not controlled, while the Sahel was underemphasized.

#### Balancing errors

Finally, we examined the effects of balancing Type 1 and Type 2 error appropriately in niche model development. This factor was examined based on the original set of occurrence data, but with different consensus methods for combining multiple replicate models in which all error was treated equally *versus* when omission error rates were prioritized over commission error rates. Overall, models with corrected (i.e., non-equal) balance of error weightings identified a dramatically broader area as suitable for LASV transmission, although the original models did not emphasize any particular area overmuch (Figure 3).

**Figure 4:**
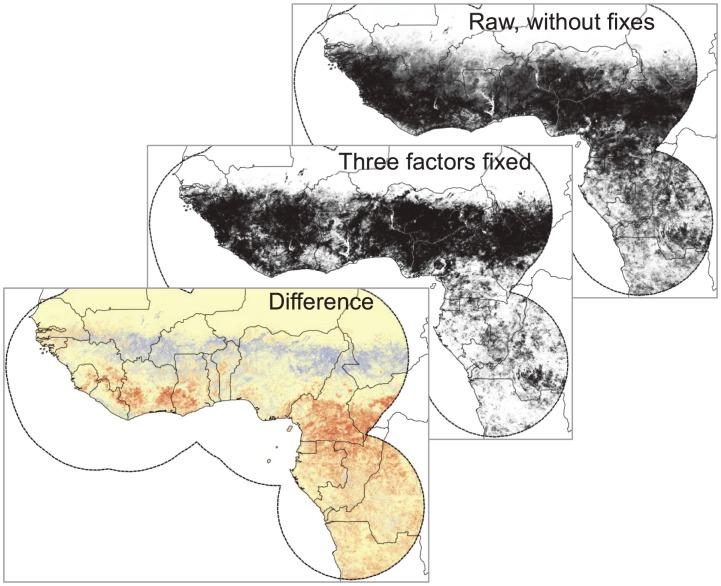
Overall effect of the three corrections explored in this paper shown as the results of the ‘raw’ models designed to mimic the original models [7] (top panel), models based on all three of the corrections together (middle), and the difference between the two (bottom). In the bottom map, red areas are those overemphasized in the raw models, while blue areas indicate underemphasis of the raw models.

Three corrections to risk mapping procedures where individually assessed in the preceding paragraphs. The comparison between the raw, unfixed, and corrected model outputs is quite instructive. Raw model outputs resemble closely the maps presented by Fichet-Calvet and Rogers [7] (see our Figures 2 and 4), emphasizing humid forest habitats across West Africa and south and east into Central Africa. In contrast, the corrected models extend considerably farther north into the more arid Sahel region, but areas of Ghana and Côte d'Ivoire show more reduced areas of suitability.

### Sierra Leone: Effects of different random subsamplings and model validation

**Figure 5:**
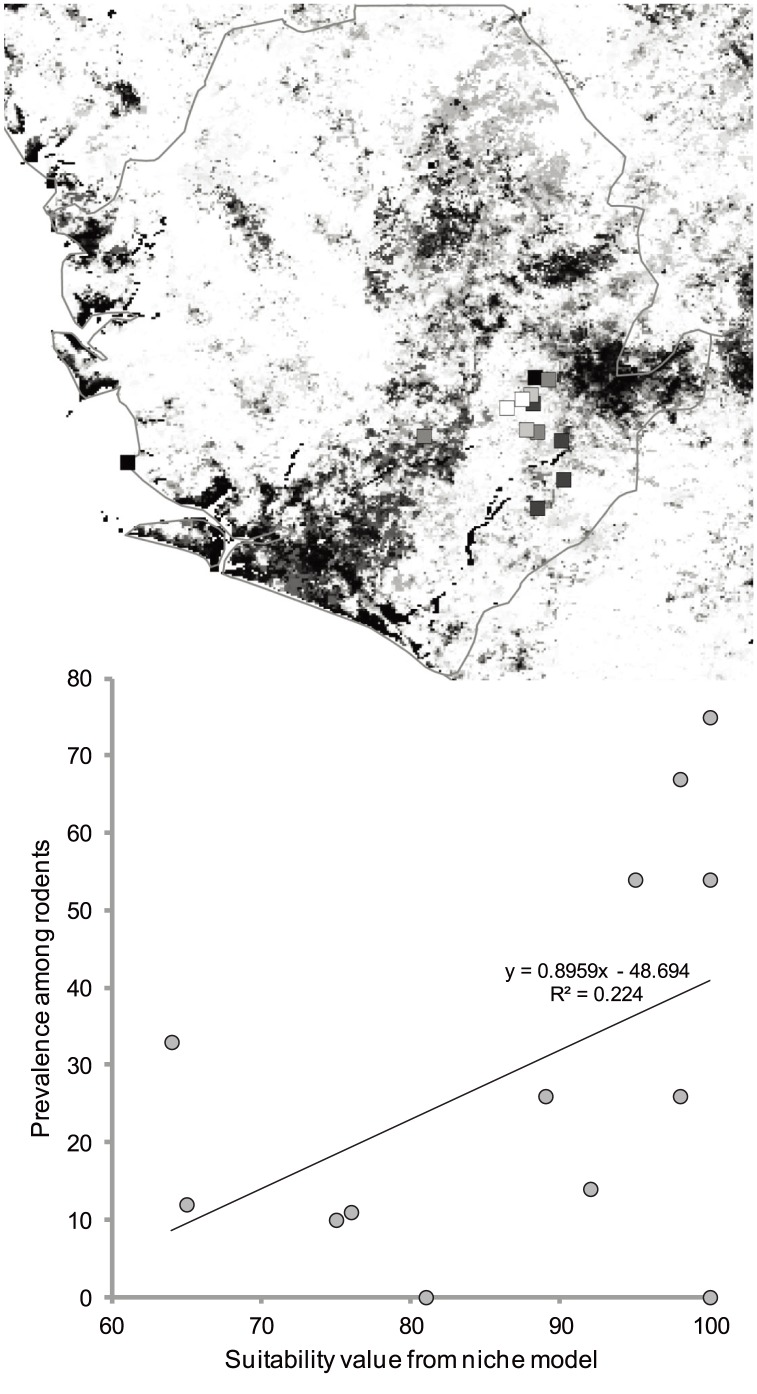
Detail of Sierra Leone from the “corrected” model shown in Figure 4. The top panel shows the modeled suitability (black = low, white = high) and LASV-infected rodent prevalences at 13 sites across the country (shading within squares, black = low, white = high). The bottom panel shows the relationship between LASV prevalences in rodents and modeled LF suitability.

Given that two of the three authors of this paper (LMM and DGB) have considerable experience with LF in Sierra Leone, we paid considerable attention to patterns of suitability that were reconstructed in that region (Figure 5). The uneven pattern of suitability in the region was intriguing, with areas of high and low suitability reconstructed across the country. Hence, we took two additional steps in exploring and understanding our models.

First, we repeated the random subsampling of 25% of the occurrence data from that region 10 times, fitting new rangewide models to see if our initial subsampling was in some way atypical or nonrepresentative of a more general tendency. The replicate analyses showed similar patterns of gaps among suitable areas across the region, so we conclude that the fragmented suitable areas shown in Figure 5 are a general pattern that is characteristic of the broader data set and not a consequence of a particular random subsampling.

Seeking an independent validation of predictions from our models (see discussion in [25]), we were frustrated by the paucity and uneven distribution of known occurrences of LF in Sierra Leone. Although a rangewide independent data set for LF is not available on which to base such a test, a smaller, more restricted data set is available from testing rodents for LASV from work by one of us (L. Moses, in prep.). Plotting LASV-infected rodent prevalences against predicted suitability from the LF models (i.e., from the map in Figure 4) shows a positive relationship in which high LASV prevalences are achieved at the highest modeled suitability levels (Figure 5; *R* ^2^ = 0.224, *P*<0.05). Hence, we see at least a local-scale confirmation that patterns of variation in modeled suitability have meaning for LASV prevalence in rodents.

## Discussion

This paper presents a series of insights into how disease transmission can and should be reconstructed across space to produce risk maps. Although we have focused on LF because of the availability of the occurrence data and initial analyses from Fichet-Calvet and Rogers [7], parallel analysis exist for the distribution of other diseases, such as monkeypox [11]. We emphasize that we in no way consider our results to be comprehensive transmission risk maps; many details remain to be addressed regarding the occurrence data (see below).

### Caveats

It may be argued that our before-and-after comparisons are not valid because we did not replicate the Fichet-Calvet and Rogers analyses exactly. Indeed, we did not replicate their procedures in two ways. First, Fichet-Calvet and Rogers took advantage of rich environmental data sets developed by Scharlemann and colleagues [26], in which multitemporal vegetation index data were processed to produce a multidimensional picture of seasonality and vegetation phenology. While we would very much have liked to use this data set in our analyses, three separate requests to the senior and corresponding authors were not answered.

Second, we did not use their same analytical approach, which was a non-linear maximum likelihood discriminant analysis. Instead, we purposefully used algorithms of known behavior [27], [28] that permitted us to assess more readily the error balance question without major programming modifications [24]. With regard to this latter point, our experience is that even very different algorithms converge on similar solutions [9], [10]. Because our “before” maps in our before-and-after comparisons (Figure 4) closely resembled those of Fichet-Calvet and Rogers [7], we believe that our manipulations were indeed effective and illustrate the effects that are the point of this article.

### The point

This set of analyses illustrates the importance of a rigorous conceptual framework of ecology and biogeography that can take into account the biases inherent in occurrence data for any modeling and mapping exercises. Careful consideration of these biases is essential in creating and interpreting distribution and risk maps [9]. A firm conceptual framework that vets data thoroughly and considers carefully how they should be incorporated into the model guides one through a series of explicit assumptions that place—to the greatest degree possible—analyses in the context of an ecological niche on a biogeographic landscape [29].

This study illustrates the point that (1) lack of quality control of occurrence data, (2) oversampling in clustered areas, and (3) inappropriate equal weighting of error components in model calibration all have macrogeographic implications for mapping disease transmission risk. Each of these factors affects estimates of risk and these effects are not randomly distributed spatially, but rather have considerable spatial autocorrelation. This autocorrelation means that these factors actually make a macrogeographic difference in the estimates that result, and that these factors must be considered within a conceptual framework that takes them into account integrally.

Indeed, if we combine the corrections to each of the three biases into a final model and compare this to the uncorrected model, we see serious geographic differences in reconstructed risk patterns (Figure 4). The area predicted as suitable for LASV transmission now appears considerably broader, and additional areas around the Dahomey Gap and in the Sahel region that the original Fichet-Calvet and Rogers maps did not include are appreciated as at risk. The reasons behind these differences are clear from Figure 1; LF occurrence points that we used to create our final models are more evenly distributed across both humid and semiarid situations than the original points used by Fichet-Calvet and Rogers, and the extreme concentration in western-most Africa is largely removed. As a result, the picture of environments suitable for LASV transmission is broadened considerably. The error balance manipulation serves to broaden this view still more. Indeed, in 2010, outbreaks of LF occurred in southern Mali and northwestern Nigeria [30], [31] (see also ProMed archive 20100519.1656), areas depicted as low risk by Fichet-Calvet and Rogers, but as high risk in our adjusted model outputs.

However, as mentioned above, even our corrected maps are far from constituting final risk maps for LF. Many nuances and additional elements would need to factored into a highly predictive map, including: (1) Detailed maps of *M. natalensis* distribution. LASV/LF occurrence data would ideally be derived from data on LASV-infected rodents, rather than humans, for the aforementioned reasons related to the magnitude of movements of rodents *versus* humans. However, understanding the distribution of *M. natalensis* has been complicated by the existence of numerous morphologically identical species and sub-species of *Mastomys* in sub-Saharan Africa, often leading to misidentifications and consequent errors in occurrence data. Fortunately, molecular assays have recently been developed to allow reliable distinction between *Mastomys* species [32]. (2) Consideration of additional human behavioral and societal factors is crucial, as they may modify risk by impacting probability of *Mastomys* occurrence and LASV infection and/or transmission to humans (e.g., poverty and socioeconomic status, levels of development, educational status, land use, housing construction) and reporting (e.g., distance to clinics and hospitals, medical personnel awareness of LF, availability of diagnostic facilities, efficiency of reporting pipelines). (3) Consideration of human genetic predisposition to LASV infection and disease is important, as data now suggest that certain genotypes in West African human populations may be protective [33]. (4) Finally, and perhaps most importantly, maps would be evaluated and tested rigorously via independent data sets, such that confidence in ‘risk’ estimates can be taken into account quantitatively. In this sense, the maps presented in this paper are not risk maps, but rather explorations of factors that affect ability to reconstruct disease transmission risk and to generate hypotheses for further field testing, verification, and map refinement.
